# Family Engagement at the Systems Level: A Framework for Action

**DOI:** 10.1007/s10995-023-03619-2

**Published:** 2023-03-13

**Authors:** Beth Dworetzky, Clarissa G. Hoover, Deborah Klein Walker

**Affiliations:** 1 Family Voices, 561 Virginia Rd, Bldg. 4, Suite 300, 01742 Concord, MA USA; 2 Tufts University School of Medicine, 136 Harrison Ave, 02111 Boston, MA USA

**Keywords:** Family engagement, Partnerships, Quality improvement, Systems-change, Children with special healthcare needs

## Abstract

While family engagement at the individual level of health care, such as families partnering with providers in decision-making about health care for an individual child has been well studied, family engagement in systems-level activities (e.g., participation in advisory and other decision-making groups, or creation and revision of policies) that impact the health services families and children receive has not. This note from the field presents a framework that describes the information and supports that help families partner with professionals and contribute to systems-level activities. Without attention to these components of family engagement, family presence and participation may be only token. We engaged an expert Family/Professional Workgroup whose members represented key constituencies and diverse geography, race/ethnicity, and areas of expertise; conducted a review of peer-reviewed publications and grey literature; and conducted a series of key informant interviews to identify best practices for supporting meaningful family engagement at the systems level. Based on an analysis of the findings, the authors identified four action-oriented domains of family engagement and key criteria that support and strengthen meaningful family engagement in systems-level initiatives. Child- and family-serving serving organizations can use this Family Engagement in Systems framework to support meaningful family engagement in the design of policies, practices, services, supports, quality improvement projects, research, and other systems-level activities.

## Significance

Health care providers, researchers, and policy makers understand the importance of engaging families at the individual and systems level where health policies are created to improve systems of care. Many articles mention that families were engaged in systems-level initiatives but provide little detail about how they provided the information and supports that families need and receive to understand their partnership roles, collaborate, and make meaningful contributions. An analysis of a literature review and key informant interviews informed the creation of a framework and the identification of key criteria to support meaningful family engagement in systems change.

## Purpose

In 1987, Surgeon General C. Everett Koop’s *Report on Children with Special Health Care Needs*^1^ proposed action steps towards achieving “comprehensive, coordinated, family-centered, community-based services for children with special needs and their families.” These action steps included the need for family/professional collaboration in program development, implementation, evaluation and policy formulation. It continues to be vitally important that individuals and families who receive services from a system of care, particularly those who experience the most disparities in healthcare, have a voice in creating or improving the policies, practices, services and supports that govern the services they receive. Several frameworks describe the continuum of family engagement in systems-level activities. For example, Carman et al. (2013) describes a continuum from consultation to involvement, to partnership and shared leadership. The Spectrum of Public Participation^2^ from the International Association for Public Participation describes roles from informing, consulting, involving, to collaborating, and empowering. However, there is no established framework that defines the components of family engagement that can assist families in moving along the continuum of family engagement. This note describes a framework for how families’ lived experience can inform and lead to meaningful contributions to systems change that can improve systems of care for all children and families.

Much of the research on family engagement has focused on the roles of family engagement in the care of an individual child (Maurer et al., 2012; Minniti et al., 2014; Berg et al., 2015; Cene et al., 2016); fewer focus on family engagement at the systems level. Yet, increasingly, child- and family-serving organizations are expected or even required to engage families in systems-level initiatives. For example, hospitals and health care organizations are engaging families as advisory board members (Dokken et al., 2021). The Maternal and Child Health Bureau (MCHB) Title V block grant guidance expects states and jurisdictions to “*Assure families and individuals are key partners in health care decision-making at all levels across the health care system and the services that support them, especially those who are vulnerable and medically underserved*.”^3^ Some states have taken legislative action to require family engagement in systems change. California Senate Bill 586 required Medi-Cal managed care programs to establish family advisory groups to “*monitor processes and outcome measures by which the plans participating in the Whole Child Model program shall be monitored and evaluated*.”^4^ Many of these organizations, however, struggle with how to engage and support the family partners with whom they collaborate to ensure they can participate and contribute to systems-level initiatives in meaningful ways.

The work presented here *identifies, for both families and professionals, the essential components of meaningful family engagement at the systems level to ensure that families have the information, support, context, and understanding of their roles and responsibilities to participate, partner, and contribute to systems-level initiatives*.

## Description

### Overall Design

This study is designed according to the processes used in prior scoping review methodologies (Hamilton et al., 2019; Hamilton et al., 2021). Unlike the former studies in which stakeholders were included in the final phases of the project, stakeholders assisted with all phases of the project, as described below.

### Stakeholder Consultation

We identified and engaged an Expert Family/Professional Workgroup from the start of the project to advise the work. The members represented key constituencies, the diverse race/ethnicity and geography of the country, and multiple areas of experience and expertise. The 15-member Workgroup met monthly throughout the 18 months of the project. Members included family leaders with lived experience and knowledge navigating health care at the systems level, as well as experience in family support and family/professional partnerships. The professionals included a pediatrician, a health plan administrator who serves children with complex needs across seven states, a Medical Director for a managed care organization, a state Title V Children with Special Health Care Needs (CSHCN) program director, and highly experienced public health policy analysts and evaluators.

The Workgroup was actively engaged in all phases of the project, from design to product development. Members assisted with the synthesis of the literature review, the development of the key informant interview guide, analysis of the findings from the literature review and interviews, identification of the key criteria, and development of the framework for assessing family engagement at the systems level to ensure that family engagement is equitable, authentic, and meaningful.

### Literature Review

We conducted a literature review, drawing from a variety of sources, within and without the maternal and child health field. This effort included peer-reviewed articles and grey literature reports that highlighted approaches to patient, family, and community engagement and provided a picture of a vibrant and increasingly evidence-based field of study. Using the query “cooperative behavior“[mesh] AND “community participation“[majr] AND (hasabstract[text] AND “humans“[MeSH Terms] AND English[lang]) in Pubmed, we identified 770 articles. The Expert Workgroup recommended an additional 21 articles. We identified an additional 7 articles linked to other articles in the review, for a total of 798, published between 2001 and 2021.^5^

Articles and reports that mentioned family engagement but did not describe how families were supported in such engagement were excluded, as our goal was to identify “how to” ensure that family engagement in systems-level initiatives is meaningful.

Using the framework described by Carman et al. (2013), two members of the project team along with several members of the Workgroup selected articles for the literature review based on the following criteria:

- The article demonstrated and/or assessed patient, family, or community engagement at the systems level.
  - Community members participated as members of a core project team.
  - Engagement efforts included training (Hawley, 2010; Robbins et al., 2016; Fraenkel et al., 2016) or other capacity-building activities, such as peer mentors (Taylor et al., 2010; NICOM, 2015) to support engagement at the systems level.
  - Community members participated as oversight/advisory council members, key informants, or in other activities that allowed for an iterative dialog between professionals and families.
  - Community members co-developed and analyzed surveys or facilitated and interpreted focus group findings.

The project team reviewed the resulting 33 articles, categorized the activities in which families were engaged, and identified the supports the families received to ensure their engagement was meaningful.

### Key Informant Interviews

To complement the literature review, the project team interviewed 19 key informants to learn about the types of system-level activities families were engaging in as well as the supports that helped families be effective contributors. Interviews also explored what changed because families were engaged and the factors that support meaningful family engagement in policy, practice, and other systems-level activities that impact health services for *all* children. The key informants, 10 family advocates who represented the diversity of the country, and 9 professionals, were recommended by the Family/Professional Expert Workgroup and by a national network of family-led organizations.

The family advocates included staff from various family-led and community-based organizations, disability advocates, and members of hospital and other advisory groups. The professionals included public health academics, public and private payers, Title V agencies, regional genetics network personnel, and pediatricians. The interview guide, informed by the findings from the literature review, was the same for both families and professionals.

Using NVivo software, research partners at Social and Behavioral Health, School of Community Health Sciences, University of Nevada, Reno performed an analysis of the interview responses.

## Assessment

### Literature Review

The project team identified the following themes in the 33 articles selected for the literature review:

#### Family Roles

Families were engaged primarily as advisory committee members or as invited attendees at other meetings and selected because they had prior experience or leadership training, mentoring and coaching, peer support, an openness to working in a collaborative setting, and willingness to listen and learn. In some cases, professionals who were also parents of children with special health care needs were asked to assume a dual role as both a family participant and professional.

#### Family Representation

A variety of articles indicated that some “systems of care” (hospitals, researchers, Title V) understood the importance of ensuring that family partners were representative of the race, ethnicity, culture, and socio-economic status of the families and populations they serve (Conway et al., 2006; Wells & Anderson, 2006; Johnson et al., 2008; Buxbaum, 2010; Mapp & Kuttner, 2013; O’Sullivan 2014; Bailey et al. 2015; AMCHP, 2016; Glader, et al. 2016; Damon 2017; Frampton et al., 2017).

#### Factors that Support Family Engagement in Systems

Wells and Anderson (2006) found that families need a “job description” to understand their role on decision-making groups. Other articles recognized that not all families can “afford” to participate unless they are compensated for their time and other expenses associated with participation (Forbat et al., 2009; O’Sullivan, 2014; Sheridan et al., 2017). Families also need to have information about the work they will do in jargon-free, plain language (Minniti et al., 2014). Additionally, Gagliardi et al., (2008) identified the importance of having a staff member to champion family engagement.

#### Impact of Family

Engagement Few articles addressed the impact of family engagement in systems-level initiatives. Carman et al. (2014) assessed impact with improved outcomes, cost savings, and family and professional satisfaction.

Plescia and Groblewski (2004) found that engaging families and assessing family needs resulted in improved policies that were more responsive to family needs. Other articles noted that providing families with the supports they needed to make a sustained contribution to the work (Wells and Anderson, 2005; Anderson and Wells, 2006; Kaehne and Catherall, 2013; Cacari-Stone et al., 2014; Carmen et al., 2014; Patient-Centered Outcomes Research Institute, 2018; Boudes et al. 2018; Mullin, 2019) resulted in policies that were more family-centered.

### Key Informant Interviews

Based on the analysis of key informant interview responses, we identified four major themes.

#### Commitment

Family leader interviewees noted the importance of having a written policy for family engagement in systems-level initiatives and of having professional staff who were champions of family engagement to set a model for any staff who might be hesitant or resistant to working with families to improve services. This finding was supported by the professional provider interviewees who shared that some of their colleagues did not always embrace the need to engage families as partners in their work, were unwilling to “check their egos at the door” and did not recognize that they did not have the same systems-issue perspective as families who received services. A pediatrician shared, “In my experience, a small minority of care team members are kind of reluctant to hear what families have to say. So, it’s important for the champion to say, no really this is OK. This is where we’re going.” In addition, the professionals stated it was easier to engage a professional who is also a parent in a dual parent-professional role or to engage advocates who help families rather than actual families. Some professionals also presumed that families were too busy taking care of their children to engage with decision-making groups. All interviewees agreed that if professionals are being paid for their time, family members should also be compensated.

#### Transparency

The family interviewees reported that their engagement activities were primarily to attend meetings, either in person, by phone, or by use of a virtual platform. Meeting types included advisory or working group meetings, or quality improvement meetings. Regardless of the type of meeting, it was important to make sure family members had a meaningful role, felt valued, and were part of the decision-making process. They noted that engagement needs to start from a shared understanding of the issue, as often family members who are asked to participate do not have the same priorities as the professionals. Often, professionals did not understand that families’ lived experiences with systems of care made them acutely aware of policies, practices, services, and supports that needed to be improved. A family leader shared, “A lot of times, the organization has already decided what’s important and fails to ask families what’s important to them. Systems can’t change if families’ priorities are not addressed.” It was noted that family-led organizations track data about the issues families experience with systems of care. These data can be shared and used to understand families’priorities for systems change. Families also noted they were often invited to meetings merely to “check a box” that a family member was present and that without defined roles and responsibilities, they felt their presence was meaningless. A family advocate noted the importance of, “making certain that families have a role and are not just a person that you’re checking off. As soon as a family feels like their voices mean absolutely nothing, they leave.”

All the key informants recognized that both families and their professional partners needed skill-building opportunities and training to ensure effective and authentic family/professional partnerships and that continued recruitment and training can help to grow and sustain family engagement within an organization. All key informants noted the importance of having connections to family-led or community-based organizations to help recruit families to participate in systems-level engagement activities, and to provide skill-building opportunities, mentoring, and other supports families might need to understand and feel confident in their partnership roles. A family advocate recognized the importance of providing families with the skills they need to participate in meaningful ways. She recommended, “A series of trainings before families engage in systems-level activities so they have an opportunity to understand how to participate and advocate for what’s important for families.” Another family leader shared, “I’m surprise at how families change throughout the training. They become clearer about their role in public policy making. And they get excited about what they can do.”

All interviewees agreed that there should be a shared understanding of the work the families and professionals would do together. A professional shared, “We wanted to level the playing field, and make lay people, especially advocates and consumers, more comfortable participating in what could be a very intimidating committee. So, we took the time at the outset to explain some very basic, objective information.” A payer noted the importance of “helping families understand the rhythm of meetings, the rules of order, and how families can access meeting minutes after a meeting is over.” She added, “We want families to be comfortable to participate. We explain things in advance and provide materials that are user-friendly, readable, not a lot of jargon. We want them to not only understand the lingo of what we do, but also be able to use it in a way that they understand it and can communicate it to somebody else.”

#### Representation

All the key informants identified the importance of engaging families. One professional stated, “They [families] bring a certain perspective that might not otherwise be represented.” Two key informants expressed concerns that committees were stacked with family members who are selected because they are easy to work with, rather than with families selected based on being representative of the race, ethnicity, geography, socioeconomic status, and gender served. A family advocate illustrated this problem, saying, “What I’ve seen happen is that they [health center staff] start off with regular patients [on an advisory board]. But then, they feel like they’re not getting what they want from the regular patients, so they start pushing those people out and replacing them with people they know and asking those people they know to go to the health center for services so that they fulfill the requirements to have the board be 51% patients. So instead of giving support to a family member or patient so they can participant, I’ve seen folks get actually pushed out.” Another informant noted her organization’s efforts to “dig deep to find people who were culturally diverse and who had different family structures” to participate in systems change.

#### Impact

All nineteen key informants agreed it is important to assess the impact of family engagement but struggled to identify criteria to use. A family advocate shared that she assessed the impact of family engagement by counting the number of policy changes that were driven by families. Other interviewees noted that successful family engagement could be assessed by having the professionals who participated as part of the initiative identify what they did differently because they listened to and used the ideas shared by the family partners. Regardless of how impact is assessed, family interviewees noted the importance of “closing the loop” with family partners—that the organization should let families know how the information families shared was used to improve existing or create new policies and services.

### Family Engagement Framework

We have developed a framework for supporting family engagement in systems-level initiatives. Our findings are summarized in Table 1: The Domains of the Family Engagement Framework and includes a definition of each domain, along with key criteria.

**Table 1:** Domains of family engagement framework

Domain	Description	Key Criteria
**Commitment**	Commitment means that the organization routinely engages families in all systems-level initiatives that affect the policies and programs that govern services for children, youth, and families.	• The organization has a written family engagement policy. • One or more staff are champions of family engagement and set an example for staff about the importance of engaging families in systems change. • The organization has a mechanism for reimbursing families for their participation.
**Transparency**	The organization clearly documents and communicates how it identifies issues faced by the children and family they serve and provides the information and supports families need to partner and contribute to systems-level activities.	• The organization provides a description of the roles and responsibilities for family partners. • Materials are provided in plain, jargon-free language. • The organization uses internal or external data or other assessment to identify family needs and priorities. • Family partners and organization staff have opportunities to develop leadership skills through training or from mentors.
**Representation**	Family partners reflect the diversity of the community served by the organization or by a specific systems-level initiative.	• The organization collaborates with a family-led or community-based organization to help recruit, train, and support families to participate in systems-level activities. • The organization ensures that family partners are representation of the demographic of the population served by the organization or specific initiative. • Consider: race, ethnicity, culture, language, geography, disability, age, gender, sexual identity, family structure, immigration status, socio-economic status, other.
**Impact**	This domain describes how the organization used families’ ideas to improve policies, programs, services, and supports.	• Family partners feel their input is valued and helps lead to change. • Family leaders participate in what decisions are made. • Agency staff are able to identify family leaders’ contributions that led to a different outcome or process than what otherwise might have occurred.

The four domains of family engagement are:

1. **Commitment** means the organization routinely engages families in all systems-level initiatives that affect the policies and programs that govern services for children, youth, and families. Key criteria that demonstrate an organization’s commitment to family engagement include having a written policy for family engagement in all systems-level activities; having one or more staff that are champions of family engagement; having mechanism for reimbursing families for their time and expertise.
2. **Transparency** means the organization clearly documents and communicates how it identifies issues faced by the children and family they serve and provides the information and supports that families need to partner and contribute to systems-level activities. Key criteria that demonstrate an organization’s transparency include: providing family partners with a description of their roles and responsibilities; ensuring meeting and other materials are written in plain, jargon-free language; using internal or external data or other assessment to identify families’ needs and priorities for change; providing family partners and staff with opportunities to develop leadership skills through training and/or from mentors.
3. **Representation** means family partners reflect the diversity of the families who are served by the organization or by the specific systems-level initiative. Key criteria that demonstrate an organization’s commitment to representation include: collaborating with a family-led or community-based organization to help recruit, train, and support families to participate in systems-level activities; ensuring that family partners are representation of the demographic of the population served by the organization or specific initiative, which may include race, ethnicity, culture, language, geography, disability, age, gender, sexual identity, family structure, immigration status, socio-economic status, other.
4. **Impact** means the organization describes how they used families’ ideas to improve policies, programs, services, and supports. Key criteria for this domain include that family partners feel their input is valued and helps lead to change; families participate in decision-making; staff can identify family leaders’ contributions and what they are doing differently because they engaged families in their work.

## Conclusion

We found excellent alignment between the criteria that support family engagement in systems in the literature and what we learned from the key informant interviews. We also learned that family and professional key informants recognized the importance of partnerships not only with individual families, but also with family-led and community-based organizations. Family-led and community-based organizations are important collaborators. These organizations can help child- and family-serving organizations build their capacity to engage families by providing workshops and other leadership-building trainings for both families and professionals. Family-led and community-based organizations can help identify, recruit, and mentor families so they feel supported in their partnership roles. Many family-led and community-based organizations track data about the issues and concerns families experience with systems of care. These data can help inform systems-level changes that improve systems of care for all children and families. Lastly, family-led and community-based organizations can help disseminate information to families so they are aware of organizations’ efforts to improve systems of care and opportunities to participate in systems change.

Each article we included in the literature review that engaged families in systems-level initiatives noted one or two supports that helped families engage in the work. However, when these criteria are compiled and sorted into the four domains of family engagement, it was clear that to engage families in efforts to improve systems of care for all families it takes more than providing compensation or materials in plain language, the most commonly cited supports. To make family engagement authentic and meaningful, and to sustain family engagement over time, both families and professionals need a variety of supports. Families need information about their roles and responsibilities, materials to ground them in their work, training to feel confident in their decision-making roles, and mentors to help them learn how to share their lived experiences in ways that can help improve systems of care for all children and families. Professionals also need and benefit from help in recruitment, training and ongoing support.

As noted, there are numerous tools for assessing family satisfaction with family-provider partnerships at the individual level. Moreover, there are existing measures and quality indicators that systems of care can use to assess improved health outcomes, patient safety, decreased costs, improved care-coordination, and other changes. However, there is no tool for assessing how well a child- and family-serving organization is doing in engaging families in efforts to improve policies, practices, services, and supports. Since family engagement is a critical component for bringing family experience into policy, practice, and research, we encourage use of the Framework for Family Engagement to ensure authentic and meaningful family engagement in systems change. As a next step, we have created the Family Engagement in Systems Assessment Tool (FESAT) that child- and family-serving organizations can use to plan, assess, and improve family engagement in systems-level activities over time.

## Family/Professional Expert Workgroup

- Deborah Allen, Ph.D. *Deputy Director, Health Promotion, Los Angeles County Department of Public Health*.
- Amal Alsamawi, *Public Health Research Assistant, University of Michigan; sibling of sister with CP, DD.*
- Christina Bethell,* *Ph.D. PhD, MPH, MBA Director of the CAHMI Professor, Bloomberg School of Public Health Johns Hopkins University*.
- Joni Bruce, *Executive Director, Oklahoma Family Network, F2F in OK;* Family Leader.
- Paul Cleary,* PhD, *Yale School of Public Health*.
- Juno Duenas, *Executive Director, Support for Families of Children with Disabilities, CA;* Family Leader.
- Merrill Friedman, *Vice President of Advocacy, Amerigroup, Wellpoint, Inc*., *VA;* sibling of an adult sister with chronic mental health issues and the grandparent of a young boy with a heart condition.
- Carolyn Gleason, *MCH Regional Consultant*.
- Bev Johnson, *President and Chief Executive Officer, Institute for Patient- and Family-Centered Care*.
- Carolyn S. Langer, MD, MPH, JD, *SVP and Chief Medical Officer Fallon Health; mother of a young adult son with autism and developmental delays.*
- Julie Lucero, *Assistant Professor, Social and Behavioral Health, School of Community Health Sciences, University of Nevada, Reno*.
- Karen Kuhlthau, Ph.D., *Associate Professor, Massachusetts General Hospital.*
- Anne Riley, Ph.D., *Professor, Johns Hopkins School of Public* Health.
- Joan Scott, *Acting Director, Division of Children with Special Health Care Needs, MCHB*.
- Renee Turchi, MD, *Assistant Professor, Drexel University, PA; Director of the Pennsylvania Medical Home Program (EPIC IC), Medical Director of Special Programs at St. Christopher’s Hospital for Children.*

## Data Availability

This article draws on an unpublished 2017 literature review; the full text and tables of this document are available upon request.

## Footnotes

1 Children with Special Health Care Needs: Surgeon General’s Report: Campaign ’87. Available at https://profiles.nlm.nih.gov/spotlight/nn/catalog/nlm:nlmuid-101584932X515-doc.

2 https://organizingengagement.org/models/spectrum-of-public-participation/

3 HRSA Health Resources & Services Administration Title V Maternal and Child Health Services Block Grant To States Program, available at https://mchb.tvisdata.hrsa.gov/uploadedfiles/TvisWebReports/Documents/blockgrantguidance.pdf.

4 https://leginfo.legislature.ca.gov/faces/billNavClient.xhtml?bill_id=201520160SB586.

5 The original literature search was conducted in 2017. In subsequent years, we searched for and reviewed new articles, employing the same selection criteria.

## Code Availability

NVivo.

## References

[CR1] Association of Maternal & Child Health Programs (AMCHP) (2016). Family Engagement in State Title V Maternal and Child Health (MCH) and Children with Special Health Care Needs (CYSHCN) Programs: A Compilation of Survey Results. Palo Alto, CA: Lucile Packard Foundation for Children’s Health. Available at http://www.amchp.org/programsandtopics/family-engagement/SiteAssets/Pages/default/Family%20Engagement%20Executive%20Summary%20v107.pdf.

[CR2] Anderson, B., & Wells, N. (2005). Families in Program and Policy: Interviews on Family Participation with State Title V Maternal and Child Health Programs. Albuquerque, NM: Family Voices. Available at http://familyvoices.org/wp-content/uploads/2016/04/Fipps_MCH_Final-1.pdf.

[CR3] Bailey, S., Boddy, K., Briscoe, S., & Morris, C. (2015). Involving disabled children and young people as partners in research: a systematic review. *Child: care health and development*, *41*(4), 505–514. 10.1111/cch.12197 25323964

[CR4] Berg, R. C., Gamst, A., Said, M., Aas, K. B., Songe, S. H., Fangen, K., & Rysstad, O. (2015). True user involvement by people living with HIV is possible: description of a user-driven HIV Clinic in Norway. *Journal of the Association of Nurses in AIDS Care*, *26*(6), 732–742. 10.1016/j.jana.2015.07.002 26255897

[CR5] Boudes, M., Robinson, P., Bertelsen, N., Brooke, N., Hoos, A., Boutin, M., & Sargeant, I. (2018). What do stakeholders expect from patient engagement: are these expectations being met? *Health Expectations*, *21*(6), 1035–1045. 10.1111/hex.12797 PMC6250871 29858529

[CR6] Buxbaum, J. (2010). *Making connections: Medicaid, CHIP, and title V working together on state medical home initiatives*. National Academy for State Health Policy: Portland, ME.

[CR7] Cacari-Stone, L., Wallerstein, N., Garcia, A. P., & Minkler, M. (2014). The promise of community-based participatory research for health equity: a conceptual model for bridging evidence with policy. *American journal of public health*, *104*(9), 1615–1623. 10.2105/AJPH.2014.301961 PMC4151933 25033119

[CR8] Carman, K. L., Dardess, P., Maurer, M., Sofaer, S., Adams, K., Bechtel, C., & Sweeney, J. (2013). Patient and family engagement: a framework for understanding the elements and developing interventions and policies. *Health Affairs*, *32*(2), 223–231. 10.1377/hlthaff.2012.1133 23381514

[CR9] Carman, K. L., Dardess, P., Maurer, M. E., Workman, T., Ganachari, D., & Pathak-Sen, E. (2014). A Roadmap for Patient and Family Engagement in Healthcare Practice and Research. Gordon and Betty Moore Foundation: Palo Alto, CA.

[CR10] Cené, C. W., Johnson, B. H., Wells, N., Baker, B., Davis, R., & Turchi, R. (2016). A narrative review of patient and Family Engagement: the “Foundation” of the medical “Home”. *Medical Care*, *54*(7), 697–705. 10.1097/MLR.0000000000000548 PMC4907812 27111748

[CR11] Conway, J., Johnson, B., Edgman-Levitan, S., Schlucter, J., Ford, D., Sodomka, P., & Simmons, L. (2006). *Partnering with patients and families to design a patient-and family-centered health care system: a roadmap for the future: a work in progress*. Bethesda, MD: Institute for Family-Centered Care.

[CR12] Damon, W., Callon, C., Wiebe, L., Small, W., Kerr, T., & McNeil, R. (2017). Community-based participatory research in a heavily researched inner city neighbourhood: perspectives of people who use drugs on their experiences as peer researchers. *Social Science & Medicine*, *176*, 85–92. 10.1016/j.socscimed.2017.01.027 PMC5438752 28135693

[CR13] Dokken, D. L., Dardess, P., & Johnnson, B. H. (2021). Key Learnings for Strenthening Partnerships: Recommendations from a National Study of Patient and Family Advisory Councils in U.S. Children’s Hospitals. Institute for Patient- and Family-Centered Care. Available at https://www.ipfcc.org/resources/IPFCC_Key_Learnings.pdf.

[CR14] Patient-Centered Outcome Research Institute (2018). Engagement Challenges, Strategies and Resources. Available at https://www.pcori.org/sites/default/files/PCORI-Patient-Stakeholder-Engagement-Challenges-Strategies-Resources-Handout-120517.pdf.

[CR15] Forbat, L., Cayless, S., Knighting, K., Cornwell, J., & Kearney, N. (2009). Engaging patients in health care: an empirical study of the role of engagement on attitudes and action. *Patient education and counseling*, *74*(1), 84–90. 10.1016/j.pec.2008.07.055 18790594

[CR16] Frampton, S., Guastello, S., Hoy, L., Naylor, M., Sheridan, S., & Johnston-Fleece, M. (2017). *Harnessing evidence and experience to Change Culture: a Guiding Framework for Patient and Family Engaged Care*. Washington D.C.: National Academy of Medicine.

[CR17] Fraenkel, L., Miller, A. S., Clayton, K., Crow-Hercher, R., Hazel, S., Johnson, B., & Singh, J. A. (2016). When patients write the guidelines: patient panel recommendations for the treatment of rheumatoid arthritis. *Arthritis care & research*, *68*(1), 26–35. 10.1002/acr.22758 PMC4715535 26545701

[CR18] Gagliardi, A. R., Lemieux-Charles, L., Brown, A. D., Sullivan, T., & Goel, V. (2008). Barriers to patient involvement in health service planning and evaluation: an exploratory study. *Patient education and counseling*, *70*(2), 234–241. 10.1016/j.pec.2007.09.009 18023129

[CR19] Glader, L., Plews-Ogan, J., & Agrawal, R. (2016). Children with medical complexity: creating a framework for care based on the International classification of Functioning, disability and health. *Developmental medicine and child neurology*, *58*(11), 1116–1123. 10.1111/dmcn.13201 27590531

[CR20] Hamilton, C., Snow, M. E., Clark, N. .Quality of patient, family, caregiver and public engagement in decision. (2019). -making in healthcare systems: a scoping review protocol. *British Medical Journal Open*, *9*, e032788. 10.1136/bmjopen-2019-032788 PMC6858224 31699750

[CR22] Hamilton, C. B., Dehnadi, M., Snow, M. E., et al. (2021). Themes for evaluating the quality of initiatives to engage patients and family caregivers in decision-making in healthcare systems: a scoping review. *British Medical Journal Open*, *11*, e050208. 10.1136/bmjopen-2021-050208 PMC8506891 34635521

[CR23] Hawley, B. (2010). Pediatric palliative and hospice care: Pennsylvania’s model of collaboration. *Pediatric nursing*, *36*(1), 61–66. Hitchen and Williamson 2015. 20361447

[CR24] Johnson, B., Abraham, M., Conway, J., Simmons, L., Edgman-Levitan, S., Sodomka, P., & Ford, D. (2008). *Partnering with patients and families to design a patient-and family-centered health care system: recommendations and Promising Practices*. Bethesda MD: Institute for Family-Centered Care.

[CR25] Kaehne, A., & Catherall, C. (2013). User involvement in service integration and carers’ views of co-locating children’s services. *Journal of health organization and management*, *27*(5), 601–617. 10.1108/JHOM-04-2012-0072 24341179

[CR26] Mapp, K. L., & Kuttner, P. J. (2013). *Partners in education: a dual capacity-building Framework for Family-School partnerships*. SEDL.

[CR27] Maurer, M., Dardess, P., Carman, K. L., Frazier, K., & Smeeding, L. (2012). Guide to patient and family engagement: Environmental scan report. Agency for Healthcare Research and Quality: Rockville, MD. Available at http://www.mirror.upsite.co.il/uploaded/files/1386_26b74f8c702c824dcfb67dd3c375a71a.pdf.

[CR28] Minniti, M. M., Abraham, M. R., & Johnson, B. H. (2014). *Individual and Family Engagement in the Medicaid Population: emerging best Practices and Recommendations*. Robert Wood Johnson Foundation: Princeton, NJ.

[CR29] Mullin, A. (2019). Impact! How consumers have shaped health system delivery reform. Community Catalyst: Boston. https://www.healthinnovation.org/resources/publications/impact-how-consumers-have-shaped-health-system-delivery-reform.

[CR30] North Carolina Institute of Medicine (NCIOM) (2015). Patient and Family Engagement: A Partnership for Culture Change. A Report of the NCIOM Task Force on Patient and Family Engagement. Morrisville, NC: North Carolina Institute of Medicine. Available at https://nciom.org/wp-content/uploads/2015/07/PFE-Report-FINAL.pdf. 10.18043/ncm.76.3.197 26510232

[CR31] O’Sullivan, M. (2014). *It takes a family: an analysis of Family Participation in Policymaking for Public Programs Serving Children with Special Health Care needs in California*. Lucile Packard Foundation for Children’s Health: Palo Alto, CA.

[CR32] Plescia, M., & Groblewski, M. (2004). A community-oriented primary care demonstration project: refining interventions for cardiovascular disease and diabetes. *The Annals of Family Medicine*, *2*(2), 103–109. 10.1370/afm.42 PMC1466659 15083848

[CR33] Robbins, M., Tufte, J., & Hsu, C. (2016). Learning to “swim” with the experts: experiences of two patient co-investigators for a project funded by the patient-centered outcomes Research Institute. *The Permanente Journal*, *20*(2), 85. 10.7812/TPP/15-162 PMC4867832 27083011

[CR34] Sheridan, S., Schrandt, S., Forsythe, L., Hilliard, T. S., & Paez, K. A. (2017). The PCORI engagement rubric: promising practices for partnering in research. *The Annals of Family Medicine*, *15*(2), 165–170. 10.1370/afm.2042 PMC5348236 28289118

[CR35] Taylor, J. E., Jones, R. M., O’Reilly, P., Oldfield, W., & Blackburn, A. (2010). The Station Community Mental Health Centre Inc: nurturing and empowering (Doctoral dissertation, Australian Rural Health Education Network). 20701415

[CR37] Extension for Scoping Reviews (PRISMA-ScR):Checklist and Explanation. Annals of 10.7326/M18-0850 30178033

[CR38] *Internal Medicine*. pp. 467–473. ISSN 0003-4819

[CR39] Wells, N., & Anderson, B. (2006). Families in Program and Policy: Interviews on Family Participation with State Title V Children with Special Health Care Needs Programs. Albuquerque, NM: Family Voices. Available at http://familyvoices.org/wp-content/uploads/2016/04/Fipps_CSHCN_Final-2.pdf.

