# Correction to: Family Engagement at the Systems Level: A Framework for Action

**DOI:** 10.1007/s10995-023-03674-9

**Published:** 2023-05-11

**Authors:** Beth Dworetzky, Clarissa G. Hoover, Deborah Klein Walker

**Affiliations:** 1 Family Voices, 561 Virginia Rd, Bldg. 4, Suite 300, 01742 Concord, MA, USA; 2 Tufts University School of Medicine, 136 Harrison Ave, 02111 Boston, MA, USA

**Correction to: Maternal and Child Health Journal**

10.1007/s10995-023-03619-2

The original version of this article unfortunately contained an error in the text.

The sentence under purpose section, “California Senate Bill 586 required Medi-Cal managed care programs to establish family advisory groups to “monitor processes and outcome measures by which the plans participating…” should read as follows:

California Senate Bill 586 required Medi-Cal managed care programs to establish family advisory groups. Family representatives from the local advisory groups also serve on the statewide stakeholder group that advises on the development of “the monitoring processes and outcome measures by which the plans participating in the Whole Child Model program shall be monitored and evaluated.

The original article has been corrected.

